# Unconscious selection drove seed enlargement in vegetable crops

**DOI:** 10.1002/evl3.6

**Published:** 2017-05-09

**Authors:** Thomas A. Kluyver, Glynis Jones, Benoît Pujol, Christopher Bennett, Emily J. Mockford, Michael Charles, Mark Rees, Colin P. Osborne

**Affiliations:** ^1^ Department of Animal and Plant Sciences University of Sheffield Sheffield S10 2TN United Kingdom; ^2^ Department of Archaeology, Northgate House University of Sheffield Sheffield S1 4ET United Kingdom; ^3^ Laboratoire Évolution and Diversité Biologique (EDB UMR5174) Université de Toulouse CNRS, ENSFEA, IRD, UPS France; ^4^ Current Address: School of Archaeology University of Oxford 36 Beaumont Street Oxford OX1 2PG United Kingdom

**Keywords:** Cereal crops, domestication, legume crops, origins of agriculture, pleiotropy, seed size, selective breeding, unconscious selection, vegetable crops

## Abstract

Domesticated grain crops evolved from wild plants under human cultivation, losing natural dispersal mechanisms to become dependent upon humans, and showing changes in a suite of other traits, including increasing seed size. There is tendency for seed enlargement during domestication to be viewed as the result of deliberate selection for large seeds by early farmers. However, like some other domestication traits, large seeds may have evolved through natural selection from the activities of people as they gathered plants from the wild, or brought them into cultivation in anthropogenic settings. Alternatively, larger seeds could have arisen via pleiotropic effects or genetic linkage, without foresight from early farmers, and driven by selection that acted on other organs or favored larger plants. We have separated these unconscious selection effects on seed enlargement from those of deliberate selection, by comparing the wild and domesticated forms of vegetable crops. Vegetables are propagated by planting seeds, cuttings, or tubers, but harvested for their edible leaves, stems, or roots, so that seed size is not a direct determinant of yield. We find that landrace varieties of seven vegetable crops have seeds that are 20% to 2.5‐times larger than those of their closest wild relatives. These domestication effect sizes fall completely within the equivalent range of 14% to 15.2‐times for grain crops, although domestication had a significantly larger overall effect in grain than vegetable crops. Seed enlargement in vegetable crops that are propagated vegetatively must arise from natural selection for larger seeds on the occasions when plants recruit from seed and are integrated into the crop gene pool, or via a genetic link to selection for larger plants or organs. If similar mechanisms operate across all species, then unconscious selection during domestication could have exerted stronger effects on the seed size of our staple crops than previously realized.

**Impact Summary**

The origins of agriculture transformed human history and have fascinated scholars for centuries. However, a number of important issues remain unresolved, including why hunter‐gatherers adopted agriculture, and how crops were domesticated to depend on people. The hallmark of grain crop domestication is a loss of natural seed dispersal mechanisms, accompanied by a range of other changes including seed enlargement. The extent to which ancient peoples knew they were domesticating crops is highly controversial. Were domestication characteristics knowingly bred into crops, or did they evolve as wild plants were repeatedly sown into cultivated soil, managed, and harvested? It is especially difficult to untangle the relative importance of these alternatives for seed size, because the large seeds that characterize domesticated grain crops may have been a breeding target for farmers interested in higher yields. We have addressed this issue by looking at the impacts of domestication on vegetable seed size. Any selective breeding of vegetables would have acted on the leaves, stems or roots consumed as food, but would not have directly affected seed size. Instead, any changes in vegetable seed size must have arisen from natural selection acting on crops in cultivated fields, or from genetic links to changes in another character like plant or organ size. Across seven vegetable species we have found strong evidence for a general enlargement of seeds due to domestication. The size of this effect falls completely within the range seen in cereals and pulse grains, raising the possibility that components of seed enlargement in these crops also evolved during domestication without deliberate foresight. This finding has important implications for crop evolution, meaning that major changes in our staple crops could have arisen without deliberate foresight by early farmers, with unconscious selection more important in the genesis of our food plants than previously realized.

## Introduction

The Neolithic origins of agriculture in multiple regions across the globe transformed human history, marking the transition from hunter‐gatherer subsistence to agricultural economies exploiting domesticated animals and crop plants (Purugganan and Fuller 2009; Larson et al. 2014). Domesticated grain crops evolved from wild plants under human cultivation, losing natural dispersal mechanisms to become dependent upon humans, and changing across a range of other traits, including loss of seed dormancy and increase in seed size (Harlan et al. 1973; Hammer 1984). Archaeobotanical evidence shows that seed enlargement during domestication was gradual, and occurred in different crops before or after the loss of dispersal (Tanno and Willcox 2006; Fuller 2007; Brown et al. 2009; Purugganan and Fuller 2011). It is widely accepted among archaeologists and biologists that at least some domestication traits evolved under unconscious selection.

Unconscious selection encompasses a number of potential mechanisms, which are united by the lack of foresight by early farmers in breeding domestication traits into their crops. Darwin's original conception was that breeding from the most valued individuals within a population, while killing those with undesirable characteristics, would bring improvements in the population without any deliberate foresight (Darwin 1859; Darwin 1868). Among the examples of domesticated animals he used to illustrate this idea, Darwin (1868) included seed enlargement in domesticated crops. The phenomenon is well known for animal domestication (Clutton‐Brock 1999), with experimental evidence suggesting that, even if selection deliberately targets particular traits, unintended changes can occur in others (e.g., in coat pigmentation or tail shape) (Trut et al. 2009), via pleiotropy or genetic linkage (Wilkins et al. 2014). However, unconscious selection may also have occurred via natural selection arising from the activities of people as they gathered plants from the wild or brought them under cultivation in anthropogenic settings (Zohary 2004; Ross‐Ibarra et al., 2007; also described as operational or automatic selection, Darlington 1956; Harlan et al. 1973). Thus, domestication traits may evolve under selection from sowing into cultivated soil, competition within crop stands and from the methods used for harvesting (Darlington 1956; Harlan et al. 1973; Rindos 1984; Ladizinsky 1987; Zohary 2004; Tanno and Willcox 2006; Weiss et al. 2006; Purugganan and Fuller 2009, 2011). Some of these mechanisms have received support from experiments (Hillman and Davies 1990; Milla and Matesanz 2017; Preece et al. 2017) and genetic analyses (Clark et al. 2006; Simons et al. 2006), but others have not (Kluyver et al. 2013; Milla and Morente‐Lopez 2015). The extent to which unconscious processes are responsible for domestication traits therefore remains highly controversial, with some authors taking the view that deliberate breeding is largely responsible for domestication trait evolution (Abbo et al. 2012, 2014a; Abbo et al. 2014b).

The discussion of deliberate breeding during domestication usually focuses on traits that are desirable when harvesting, handling or processing seeds, following the argument that hunter‐gatherers had a deep botanical knowledge and understood the value of selecting for these traits (Abbo et al. 2011, 2014a). Distinguishing these potential mechanisms from unconscious processes is particularly challenging for grain crops, since seed size is an important component of yield and deliberate selective breeding for harvest traits could be responsible for the observed changes. However, this problem is neatly circumvented in vegetable crops, where roots, stems, or leaves are harvested for human consumption, rather than seeds. In the case of crops like carrot or lettuce, seeds are planted by farmers, but selective breeding for yield acts on other parts of the plant, such as roots (e.g., carrot) or leaves (e.g., lettuce). In the case of tuber crops, farmers rarely (if ever) plant seeds deliberately, and crops are propagated vegetatively by planting tubers or stem cuttings.

Vegetable crops therefore offer a unique opportunity for testing the hypothesis that seed enlargement during crop domestication was driven by unconscious selection mechanisms. In vegetable crops, these could include: (1) pleiotropic or genetic linkage effects arising indirectly from selection for increasing plant or organ size; and (2) natural selection arising from plant cultivation, and acting on seeds and seedlings. Deliberate selection could conceivably have occurred in the vegetable crops propagated by seeds if: (3) larger seeds were easier to sow or perceived by farmers as being a higher quality than smaller seeds. However, any domestication effect on seed size in vegetable crops is unlikely to have arisen from: (4) deliberate selective breeding for seed harvest traits. In this article, we evaluate these four potential mechanisms by comparing the effects of domestication on the seeds of cereal (annual grass), pulse (grain legume), and vegetable crops propagated vegetatively or via seeds. We show evidence of seed enlargement in domesticated vegetable crops, whose magnitude is comparable with that in cereals and pulses, and is likely explained by unconscious selection.

## Methods

### SPECIES SELECTION

We focused on crop species thought to have been domesticated in antiquity (Ugent et al. 1982; Piperno et al. 2000; Lebot 2009; Piperno et al. 2009; Zohary et al. 2012). For seed crops, we used a range of cereals and pulses domesticated in different parts of the world (Tables 1 and 2). For vegetable crops, we looked both for species that are typically grown from seed, and species that are vegetatively propagated (Table 3). Fruit crops were not included in these comparisons.

**Table 1:** Cereal crops and sources of data used in the seed size comparison

Common name	Centre of domestication	Domesticated landrace	Wild relative(s)	Literature sources	Data/seed sources
Barley	Western Asia	*Hordeum vulgare* L.	*Hordeum spontaneum* C. Koch	(Zohary *et al*. 2012)	GRIN, SID, IPK
Einkorn wheat	Western Asia	*Triticum monococcum* L.	*Triticum boeoticum* Boiss.	(Zohary *et al*. 2012)	GRIN
Emmer wheat	Western Asia	*Triticum dicoccon* (Schrank) Schübl.	*Triticum dicoccoides* (Koern.) G. Schweinfurth	(Zohary *et al*. 2012)	GRIN, SID, IPK
Foxtail millet	China	*Setaria italica* (L.) Beauv.	*Setaria viridis* (L.) Beauv.	(Zohary *et al*. 2012)	GRIN
Maize	Mesoamerica	*Zea mays* L.	*Zea mexicana* (Schrad.) Kuntze, *Zea mays* subsp. *parviglumis* H.H. Iltis & J. F. Doebley	(Hufford *et al*. 2012)	GRIN
Oats	Western Asia (Europe?)	*Avena sativa* L.	*Avena sterilis* L.	(Zohary *et al*. 2012)	GRIN
Pearl millet	Sub‐Saharan Africa	*Pennisetum glaucum* (L.) R. Br.	*Pennisetum violaceum* (Lam.) Rich.	(Brunken *et al*. 1977; Harlan 1992)	GRIN
Rice	China	*Oryza sativa* L.	*Oryza rufipogon* Griff. (inc. *Oryza nivara* S.D.Sharma & Shastry)	(Fuller 2007; Zohary *et al*. 2012)	GRIN, IRRI, AusPGRIS
Rye	Western Asia	*Secale cereale* L.	*Secale vavilovii* Grossheim	(Zohary *et al*. 2012)	GRIN
Sorghum	Sub‐Saharan Africa	*Sorghum bicolor* (L.) Moench	*Sorghum arundinaceum* (Desv.) Stapf	(Aldrich and Doebley 1992; Wasylikowa *et al*. 1997; Zohary *et al*. 2012)	GRIN

All crops are annual grass (Poaceae) species exploited for their seeds. For each species, we list its geographical centre of domestication, Linnaean names of the domesticated landrace and its wild relative(s), literature sources to support the choice of wild relative(s) in each case, and use the taxonomy of Clayton et al. (2002). Sources of data and materials are listed with the following abbreviations: the USDA GRIN/NPGS database (GRIN); the Seed Information Database (SID) of The Royal Botanic Gardens, Kew; IPK Gatersleben (IPK); the International Rice Research Institute (IRRI); and the Australian Plant Genetic Resources Information System (AusPGRIS).

**Table 2:** Pulse crops and data used in the seed size comparison

Common name	Centre of domestication	Domesticated landrace	Wild relative(s)	Literature sources
Chickpea	Western Asia	*Cicer arietinum* L.	*Cicer reticulatum* Ladiz.	(Zohary *et al*. 2012)
Common Bean	Mesoamerica	*Phaseolus vulgaris* L.	*Phaseolus vulgaris* var. *aborigineus* (Burkart) Baudet	(Gepts and Debouck 1991)
Cowpea	Sub‐Saharan Africa	*Vigna unguiculata* (L.) Walp.	*Vigna unguiculata* subsp. *dekindtiana* (Harms) Verdc.	(Lush and Evans 1981)
Lentil	Western Asia	*Lens culinaris* Medik.	*Lens culinaris* subsp. *orientalis* (Boiss.) Ponert	(Zohary *et al*. 2012)
Lima Bean	Mesoamerica	*Phaseolus lunatus* L.	*Phaseolus lunatus* L.	(Serrano‐Serrano *et al*. 2012)
Mung Bean	India	*Vigna radiata* (L.) R. Wilczek	*Vigna radiata* var. *sublobata* (Roxb.) Verdc.	(Fuller 2007; Kang *et al*. 2014)
Pea	Western Asia	*Pisum sativum* L.	*Pisum sativum* L. (including subsp. *elatius* (M.Bieb.) Asch. & Graebn.)	(Zohary *et al*. 2012)
Peanut	South America	*Arachis hypogaea* L.	*Arachis monticola* Krapov. & Rigoni	(Grabiele *et al*. 2012)
Soybean	China	*Glycine max* (L.) Merr.	*Glycine max* subsp. *soja* (Siebold & Zucc.) H. Ohashi	(Kim *et al*. 2010)

All are annual legume (Fabaceae) species exploited for their seeds (pulses). For each species, we list its geographical center of domestication, Linnaean names of the domesticated landrace and its wild relative(s), literature sources to support the choice of wild relative(s) in each case, and use the taxonomy of ILDIS (2005). All data and materials were sourced from the USDA GRIN/NPGS database (GRIN) or the Australian Plant Genetic Resources Information System (AusPGRIS) (Mung Bean only).

**Table 3:** Vegetable crops and sources of data used in the seed size comparison

Common name	Propagule	Centre of domestication	Domesticated landrace	Wild relative(s)	Literature sources	Data/material sources
Beet	S	Western Asia, Mediterranean, Europe?	*Beta vulgaris* L. subsp. *vulgaris*	*Beta vulgaris* L. subsp. *maritima* (L.) Arcang.	(Zohary *et al*. 2012)	GRIN, IPK
Carrot	S	Western Asia, Mediterranean?	*Daucus carota* L.	*Daucus carota* L.	(Zohary *et al*. 2012)	GRIN
Lettuce	S	Western Asia, Mediterranean?	*Lactuca sativa* L.	*Lactuca serriola* L.	(Zohary *et al*. 2012)	GRIN
Parsnip	S	Western Asia, Mediterranean, Europe?	*Pastinaca sativa* L.	*Pastinaca sativa* L.	(Zohary *et al*. 2012)	GRIN
Cassava	T	South America	*Manihot esculenta* Crantz	*Manihot esculenta* Crantz spp *flabellifolia*	(Olsen & Schaal 1999)	EMBRAPA, (Pujol *et al*. 2005)
Potato	T	South America	*Solanum tuberosum* subsp. *tuberosum* L., *Solanum tuberosum* subsp. *andigena* (Juz. & Bukasov) Hawkes, *Solanum stenotomum*, *Solanum phureja*	*Solanum brevicaule* complex (*S. brevicaule* Bitter, *S. bukasovii* Juz. ex Rybin, *S. canasense* Hawkes, *S. candolleanum* P. Berthault, *S. gourlayi* Hawkes and *S. spegazzinii* Bitter), *Solanum acaule* Bitter	See Suppl. Discuss. 1	GRIN, IPK
Sweet Potato	T	South America	*Ipomoea batatas* (L.) Lam.	*Ipomoea trifida* (Kunth) G. Don	(Kyndt *et al*. 2015)	GRIN, CIP

For each species, we list the type of propagule used for cultivation (T, tuber or S, seed), its geographical center of domestication, Linnaean names of the domesticated landrace and its wild relative(s), and literature sources to support the choice of wild relative(s) in each case. Sources of data and materials are listed with the following abbreviations: the USDA GRIN/NPGS database (GRIN); IPK Gatersleben (IPK); EMBRAPA Genetic Resources and Biotechnology Centre (EMBRAPA); and the International Potato Centre (CIP).

We note that some species now regarded as vegetable crops may in earlier times have been seed crops, and subject to different selection pressures. Of the crops considered here, archaeological evidence suggests that the carrot in Europe and lettuce in Egypt may have been used millennia ago as seed crops (Andrews 1949; deVries 1997; Iorizzo et al. 2013). However, such use represents a negligible part of their evolutionary history of domestication. The literature does not indicate that any of the other vegetable crop species examined in this study were ever grown for seed (Ugent et al. 1982; Smartt and Simmonds 1995; Lebot 2009; Zohary et al. 2012).

The range of crops was limited by the species for which seed mass data is available, especially in vegetatively propagated crops where seeds are used more rarely. All three vegetatively propagated species tested originate from South America: efforts to obtain true seed of taro, *Colocasia esculenta* (L.) Schott, which was domesticated in the Asia‐Pacific region, were unfortunately unsuccessful.

In the case of neopolyploid crops, the immediate progenitor of the same ploidy level was used, such as *Triticum dicoccoides* Koern. for emmer wheat and *Arachis monticola* Krapov. & Rigoni for peanut (Seijo et al. 2007). Genome donors were not considered progenitors, as they do not represent the plants that early famers chose to cultivate. Therefore, no progenitor species is included for bread wheat, *Triticum aestivum* L., a hexaploid believed to have been arisen in cultivation.

### DATA SOURCES

For each species of interest, we initially used a custom script to download data with permission from the USDA GRIN germplasm database. Where there were multiple mass measurements for one accession, these are summarized as the arithmetic mean, so that each datum represents a single accession. Where there were insufficient data to allow a comparison, seed accessions were ordered and weighed. Seed crops were largely obtained from GRIN, except for mung bean (*Vigna radiata* (L.) R. Wilczek), which came from the Australian AusPGRIS collection. Additional sources of seed crop data and materials are listed in Tables 1, 2. Data and seeds of root crops were sourced from:

- The USDA GRIN/NPGS database (http://www.ars-grin.gov/npgs/).
- IPK Gatersleben (http://gbis.ipk-gatersleben.de).
- The International Potato Centre (CIP) in Peru (http://cipotato.org/).
- EMBRAPA in Brazil (http://tirfaa.cenargen.embrapa.br)
- Cassava seed masses collected by Pujol et al. (2005).

Sufficient true seed or true seed mass data were sourced for seven vegetable crop species to compare wild and domestic forms (Table 3). Of these, four are crops typically grown from seed, and three are vegetatively propagated tuber crops (see Table 3).

### SEED MASS COMPARISONS

The domestic forms were all landrace accessions, to exclude any effect of modern commercial breeding. For all crops, only wild and landrace seeds that were collected from the broad region in which the crop originated were included, to limit the inclusion of feral accessions of varieties developed by modern breeding.

Both the seeds and the seed mass data available for beet are actually seed capsules, each containing one or two seeds in a tough, woody structure. We therefore dissected capsules after soaking in water for half an hour to soften them, and weighed 5–10 true seeds per accession.

**Figure 1:**
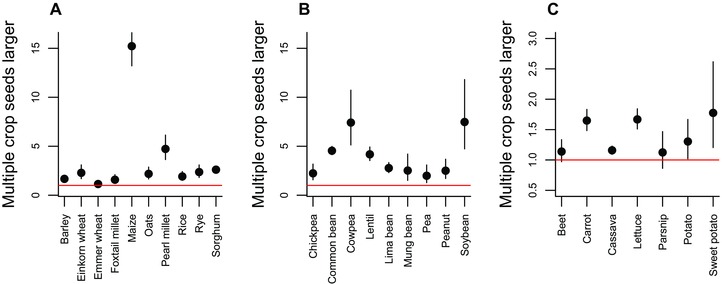
Comparisons of seed mass between landraces and wild accessions of (A) cereals (annual grass crops), (B) pulses (grain legumes), and (C) vegetables. The seed mass in domesticated crop plants is expressed as a multiple of that in wild plants (i.e., a value of two indicates a twofold increase in seed mass under domestication). Points represent mean ± 95% confidence interval and the red line denotes a value of 1.0 (i.e., no effect of domestication).

Seed masses typically follow a log‐normal distribution (Leishman et al. 1995), and so data were log‐transformed prior to analysis. Linear‐mixed effects models were fitted for cereals, pulses, and vegetables independently, using the lme4 package in R. In each case, domestication/improvement status was fitted within taxon, with country of origin fitted as a random effect. The effect sizes and their 95% confidence intervals in Figure 1 were calculated from the fitted lmer model. As the *P*‐values for the fixed effects in lmer models are typically anti‐conservative (too small) we refitted the models in MCMCglmm and used the *P*‐values from the posterior distribution. We used 100,000 iterations with a thinning interval of 50.

## Results

We first established a baseline for the magnitude and generality of increases in seed mass by comparing seed masses from wild and landrace accessions of important cereal and pulse crops (Tables 1 and 2). Landraces are locally adapted, traditional varieties of domesticated crops, which have not been subjected to intensive improvement. Seeds in these landrace forms of the crops were between 14% heavier and 15.2 times heavier than seeds from their respective wild progenitors (Fig. 1A and B). These effects were highly significant (*P* < 0.0005 in all cases) for all species except emmer wheat. We also compared seed masses for three major vegetable crops that are propagated vegetatively and harvested as tubers (potato, cassava, sweet potato), and four vegetables that are sown as seeds, but in which leaves or roots are harvested (leaf and root beet, carrot, lettuce, and parsnip) (Table 3). In five out of seven vegetable species, landrace seeds had significantly larger masses than their wild counterparts (Fig. 1C; carrot, cassava, lettuce *P* < 0.0005, sweet potato *P* < 0.007, potato *P* < 0.04), with the exceptions of beet and parsnip. This result was robust to various assumptions made about the wild progenitor(s) of domestic potato (see Supplementary Material 1 for details).

We next ascertained the provenance of the vegetable seeds, which confirmed that the observed differences could not be explained by an environmental effect. This is because seeds had been regenerated in common garden conditions in the field or glasshouse in almost all cases (see Supplementary Material 2 for details). Where original wild collections were used, these were sometimes slightly smaller in mass with a greater variance than those from common gardens, but the results were robust to the exclusion of this wild‐collected material (see Supplementary Material 2 for details).

Next, we carried out sensitivity tests to confirm that any mistakes in the classification of accessions as wild or landrace would make our estimates of domestication effect size conservative. For example, it is likely that some of the accessions included in the analysis as wild are actually feral (i.e., naturalized populations of the domesticated crop), or the result of interbreeding of wild populations with cultivated varieties. In these cases, the seed size characteristics of the domesticated crop would be mistakenly classified with the wild accessions. Our analysis shows that any such misclassification would diminish the estimated difference between wild and domesticated forms (see Supplementary Material 3 for details).

Finally, since the accessions within each species are not phylogenetically independent, we also used a sign test to make a highly conservative statistical comparison. In the vegetable crops examined, our results showed that all the landrace accessions had a larger average seed mass than their closest wild relatives. The probability of the crops having larger seed mass in all seven pairs, if there were no underlying difference was (0.5)^7^, or 0.008. This highly conservative result strengthens our conclusion that unconscious selection acted on seed size during vegetable crop domestication.

## Discussion

The observed effects of domestication are of particular interest for vegetatively propagated tuber crops, since artificial selection could not directly act on seed collected and replanted by cultivators. There are two possible mechanisms for the evolutionary change in seed size. First, volunteers may grow from seed and be incorporated in the crop gene pool, allowing natural selection to act directly on seed traits affecting natural dispersal, germination, seedling growth, and survival in cultivated environments (i.e., Hypothesis 2) (Pujol et al. 2005). Ethnographic evidence for several vegetatively propagated crops supports this hypothesis (see Supplementary Material 4 for details). If selection is able to act on volunteer seedlings, why might it favor larger seeds? First, larger true seeds of tuber crops germinate faster and more reliably than small seeds (Martin and Cabanill 1966; Strauss et al. 1979; Bhatt et al. 1989). The broader ecological literature also indicates that the larger seedlings emerging from larger seeds compete more strongly and are more likely to survive than smaller seedlings (Westoby et al. 1992). Fast germination and large initial size may be especially advantageous when in competition with a crop growing from tubers, which can store many times more resources than do seeds. However, in the cases of crops propagated by seeds, including vegetables and grain crops, these traits would also benefit individuals in competition with smaller‐seeded genotypes of the same species (Harlan et al. 1973).

The second possible mechanism is that seed enlargement may arise indirectly from selection that acts on plant or organ size, via a pleiotropic or genetic linkage effect (i.e., Hypothesis 1). For instance, true seed weight in potatoes is genetically correlated with tuber yield and harvest index (Dayal et al. 1984). Such empirical relationships among leaf, stem, and inflorescence size are well established among wild plant species, and are underpinned by allometric and developmental constraints (Primack 1987; Midgley and Bond 1989). An allometric link between plant size and seed size is expected from theory because maximum seed size is constrained by the size of terminal branches (Aarssen 2005; Grubb et al. 2005). Similarly, developmental constraints may prevent seed number from increasing in proportion to available resources (Vega et al. 2001), thereby pushing extra resources into larger seeds. These mechanisms may act in cassava, where seed capsules have a fixed three seeds per capsule (FAO 2007), and sweet potato, where capsules are limited to at most four seeds, and normally hold one or two (Martin and Cabanill, 1966). In contrast, potato often sets over 100 seeds per berry, and the number varies within and between cultivars (Almekinders et al. 1995). Similar selection mechanisms are also likely in vegetable crops propagated via seeds (beet, carrot, lettuce, parsnip) and in grain crops, although the importance of direct selection on seedling traits is likely to be greater in these species (i.e., Hypotheses 2 and 3).

Overall, the increase in seed size in vegetable crops fell entirely within the range observed in cereals and overlapped with that in pulses, implying that the effects of unconscious selection are general and would be sufficient to account for much of the observed effect of domestication in seed crops. The importance of gigantism in grain crop domestication has long been recognized, whereby domesticated plants are larger, fleshier, and more robust than their wild progenitors because seeds, leaves, stems, roots, and other organs are all enlarged (Schwanitz 1966; Evans 1993). Recent work has shown that this gigantism is an important explanation for the greater yield of domestic landraces than their wild progenitors in cereals, pulses, sunflower, tomato, chard, and cabbage (Milla and Matesanz 2017; Preece et al. 2017). In fact, these effects of domestication on plant size are correlated across species with those on seed size, such that large domestication effects on plant size mirror large effects on seed size (Milla and Matesanz 2017), providing indirect evidence to support Hypothesis 1. However, there has been little progress in understanding the mechanisms responsible for gigantism during domestication. If we make the conservative assumption that there is no general difference between seed and vegetable crops in the genetic architecture of plant and seed size, then the generality of seed enlargement across both groups implies that pleiotropic, genetic linkage, or allometric effects may play important roles in enlarging seeds in grain crops, as well as in vegetables.

Overall, however, the seed enlargements associated with domestication were larger in cereals and pulses than in vegetables (Mann–Whitney U test on increase ratios: U = 16, *P* < 0.002). This suggests the additional effects in seed crops of deliberate human selection for larger seeds as a desirable harvest trait (Hypothesis 4), or natural selection that is more specifically related to harvesting or grain processing, than to seedling performance (Hypothesis 2). In addition, the mechanisms acting on seedlings (Hypotheses 2 and 3) would have more opportunities to operate in grain crops that are grown annually from seed than on vegetatively propagated crops where sexual recruitment is infrequent.

In conclusion, our results showed that seed mass has increased during domestication in a number of vegetable crops where seed is not normally harvested, including some that are propagated vegetatively. This effect is most likely to arise from natural selection for larger seeds on the occasions when plants grow from seed and are integrated into the crop gene pool, or via a pleiotropic effect or genetic linkage to selection for larger plants or organs. The sizes of these domestication effects for vegetables fall completely within the equivalent range for grain crops. This finding implies that unconscious selection explains at least part of the domestication effect in these crops too, exerting stronger, and more general effects on seed size during domestication than have been previously realized.

### AUTHOR CONTRIBUTIONS

T.A.K. conceived the idea for this work and collected the data; C.B. and E.J.M. collected data for beet, and B.P. provided the data for cassava; T.A.K. and M.R. analyzed the data; T.A.K. and C.P.O. designed the project and wrote the article; all authors interpreted the data and edited the text.

## Supporting information

Supplementary Material
