# Supplementary material for: Unconscious selection drove seed enlargement in vegetable crops
Source: Evol Lett. 2017 May 9;1(2):64–72. doi: 10.1002/evl3.6 (PMC6121828; doi:10.1002/evl3.6)
Supplement: Supplementary file 1 — Supplementary Material [file EVL3-1-64-s001.docx]

Supplementary Material

## Supplementary Material 1

**The wild progenitors of domesticated potatoes.** Wild and cultivated potatoes (*Solanum* sect. *Petota*) include an array of introgressing species with a range of ploidy levels, and treatments of their taxonomy differ substantially (1). In our comparison, the landrace accessions included representatives of the subspecies cultivated worldwide, *Solanum tuberosum* subsp. *tuberosum* L., as well as Andigena potatoes, *S. tuberosum* subsp. *andigena* (Juz. & Bukasov) Hawkes, and two diploid cultivated species, *S. stenotomum* and *S. phureja*. The wild accessions included six species from the *Solanum brevicaule* complex, from which cultivated potatoes are thought to have originated (2): *S. brevicaule* Bitter, *S. bukasovii* Juz. ex Rybin, *S. canasense*  Hawkes, *S. candolleanum* P. Berthault, *S. gourlayi* Hawkes and *S. spegazzinii* Bitter. We also included four accessions of the wild species *S. acaule* Bitter, although it is probably more distantly related to cultivated potatoes, as a control for an effect of polyploidy on seed size: like the predominant cultivated potatoes and Andigena potatoes, *S. acaule* is a tetraploid (3).

The values given in Fig. 1C for the difference in potato seed mass between wild and domestic accessions are calculated from all the values available, including both the *Solanum brevicaule* complex and *S. acaule*. However, the difference is insensitive to assumptions about the wild progenitor taxa, and remains significant both if we exclude *S. acaule*, thought to be more distantly related (p=0.0038, Mann-Whitney U test), and if we compare only the likely tetraploids, *S. acaule* and *S. tuberosum* (p=0.036, Mann-Whitney U test).

## Supplementary Material 2

**Comparison of seeds from common gardens with those collected in the wild.** We ascertained the provenance of seeds by correspondence with the curators of germplasm collections.

All seed from IPK Gatersleben was regenerated either in common field conditions at Gatersleben or, for potatoes, in greenhouse conditions in Groß Lüsewitz. The sweet potato accessions ordered from GRIN were all seed lots, which had been regenerated in greenhouse conditions in Georgia, USA.

The carrot and parsnip seed data obtained from GRIN were a mixture of ‘original lots’ directly obtained by collection and ‘increase lots’ from regeneration in Iowa, USA. Seed from increase lots is on average slightly larger (p<0.000001, 0.0058 for carrot, parsnip; paired t-test) and has smaller variance (p<0.00001, 0.0017; Bartlett's test on log-transformed data). However, the difference between wild and landrace seeds was significant when only the data from increase lots were considered, both for carrot (p<0.000001, t-test on log-transformed data) and parsnip (p<0.03, Mann-Whitney U test).

The beet and lettuce seed data obtained from GRIN were likewise a mixture of directly collected and increased lots. The beet seeds from increase lots were on average smaller (t=11.9, p<0.000001; paired t-test) and less variable in size (t=64.3, p<0.000001; Bartlett's test). In lettuce, seeds did not significantly change in size (t=1.06, p=0.28; paired t-test), but were considerably less variable, due to the presence of a few implausible outliers in the seed masses recorded on collection (t=911, p<0.000001; Bartlett's test). The data was insufficient to confirm a difference between wild and landrace accessions from the masses of increased seed.

The wild and landrace cassava seed were collected in separate sites: wild seed was from a site in Rondonia, Brazil, while landrace seed was from a separate site in Rondonia, and two further sites in French Guiana (4). Some additional seed mass data was obtained from EMBRAPA, Brazil, in which sample the wild and domestic accessions did not differ (U=90.5, p=0.440), but the statistical power was limited, as this included only five domestic accessions. The wild accessions from EMBRAPA had significantly larger seeds than both the wild (U=138, p<0.000001) and landrace (U=837.5, p<0.000001) accessions collected by Pujol *et al.* (4). In summary, environmental effects are possible in the case of cassava, since seeds were collected from a number of different locations. We attempted to control for these in the main analysis by including country of origin as a random effect.

**Supplementary Material 3**

**The effect of misclassifying feral plants from cultivated lineages as wild.** In some cases, the classification of seed accessions as either wild or landrace may not be entirely reliable. However, this would have the effect of diminishing rather than augmenting differences, as we show below.

In lettuce, for example, some accessions of the domestic taxon, *Lactuca sativa* L., were listed as wild material. These were excluded from the main analysis, but their seed masses were more similar to landrace accessions (t-test on log-transformed data: t=1.14, p=0.25) than to accessions of the wild taxon (*Lactuca serriola* L.; t=4.25, p<0.00001), suggesting that they represent feral plants from cultivated lineages.

It is likely that some of the other accessions included in the analysis as wild are also feral, or may have interbred with cultivated varieties. However, since any such confusion would reduce the differences between wild and landrace accessions, and since there was a significant difference in most cases, it does not affect conclusions.

**Supplementary Material 4**

**Ethnographic evidence for the recruitment of seedlings in tuber crops.** Cassava crops grown under swidden cultivation often include volunteer seedlings from dormant seeds in the soil, and farmers following traditional practices include these volunteers in later vegetative propagation (5-7), influencing the crop's evolution (4, 8). Some Andean farmers deliberately save and plant potato true seed, in part to eliminate viral diseases affecting the tubers (9, 10), while traditional farming practices also make recruitment of volunteer seedlings likely, as fields typically contain a mixture of varieties rather than a clonal monoculture (11, 12). There are a few reports of the preservation of volunteer seedlings of sweet potato in New Guinea, a secondary centre of diversity (13, 14), of taro (*Colocasia esculenta* (L.) Schott; a vegetatively propagated crop domesticated in the Asia-Pacific region) in Vanuatu (15, 16), and of ensete (*Ensete ventricosum* (Welw.) Cheesman; a multi-purpose African crop) in Ethiopia (17). In contrast, there is no evidence that traditional cultivation of three minor Andean tuber crops (Oca, *Oxalis tuberosa* Molina; Ulluco, *Ullucus tuberosus* Caldas; Mashua, *Tropaeolum tuberosum* Ruiz & Pav.) recruits volunteer seedlings (18, 19), although the use of plants originating from seedlings in the past has been posited as an explanation for the observed diversity in all three species (19-21).

**Supplementary References**

1 Ovchinnikova A *et al.* (2011) Taxonomy of cultivated potatoes (Solanum section Petota: Solanaceae). *Bot J Linn Soc* 165: 107-155, doi:10.1111/j.1095-8339.2010.01107.x.

2 Spooner DM, McLean K, Ramsay G, Waugh R, Bryan GJ (2005) A single domestication for potato based on multilocus amplified fragment length polymorphism genotyping. *Proc Natl Acad Sci USA* 102: 14694-14699, doi:10.1073/pnas.0507400102.

3 Iwanaga M, Freyre R, Watanabe K (1991) Breaking the crossability barriers between disomic tetraploid *Solanum acaule* and tetrasomic tetraploid *Solanum tuberosum*. *Euphytica* 52: 183-191, doi: 10.1007/Bf00029395.

4 Pujol B *et al.* (2005) Evolution under domestication: contrasting functional morphology of seedlings in domesticated cassava and its closest wild relatives. *New Phytol* 166: 305-318, doi:10.1111/j.1469-8137.2004.01295.x.

5 Salick J, Cellinese N, Knapp S (1997) Indigenous diversity of cassava: generation, maintenance, use and loss among the Amuesha, Peruvian Upper Amazon. *Econ Bot* 51: 6-19, doi: 10.1007/Bf02910400.

6 Elias M, Rival L, McKey D (2000) Perception and management of Cassava (*Manihot esculenta* Crantz) diversity amongst Makushi Amerindians of Guyana (South America). *Journal of Ethnobiology* 20: 239-265.

7 Pujol B *et al.* (2002) Germination ecology of cassava (*Manihot esculenta* Crantz, Euphorriaceae) in traditional agroecosystems: seed and seedling biology of a vegetatively propagated domesticated plant. *Econ Bot* 56: 366-379, doi: 10.1663/0013-0001(2002)056[0366:Geocme]2.0.Co;2.

8 Pujol B, David P, McKey D (2005) Microevolution in agricultural environments: how a traditional Amerindian farming practice favours heterozygosity in cassava (*Manihot esculenta* Crantz, Euphorbiaceae). *Ecol Lett* 8: 138-147, doi:10.1111/j.1461-0248.2004.00708.x.

9 Malagamba JP & Monares A (1988) *True potato seed: past and present uses*. International Potato Center (Lima, Peru).

10 Quiros CF *et al.* (1992) Increase of potato genetic resources in their center of diversity: the role of natural outcrossing and selection by the Andean farmer. *Genet Resour Crop Ev* 39: 107-113, doi:10.1007/BF00051229.

11 Jackson MT, Hawkes JG, Rowe PR (1980) An ethnobotanical field study of primitive potato varieties in Peru. *Euphytica* 29: 107-113, doi: 10.1007/Bf00037254.

12 Johns T & Keen SL (1986) Ongoing evolution of the potato on the Altiplano of western Bolivia. *Econ Bot* 40: 409-424, doi: 10.1007/Bf02859652.

13 Yen DE (1960) The sweet potato in the Pacific: The propagation of the plant in relation to its distribution. *The Journal of the Polynesian Society* 69: 368-375.

14 Bulmer R (1965) Beliefs concerning the propagation of new varieties of sweet potato in two New Guinea Highlands societies. *The Journal of the Polynesian Society* 74: 237-239.

15 Caillon S & Lanouguère-Bruneau V (2005) Gestion de l’agrobiodiversite dans un village de Vanua Lava (Vanuatu) : strategies de selection et enjeux sociaux. *Le Journal de la Societe des Oceanistes*, 129-148.

16 Caillon S, Quero-Garcia J, Lescure JP, Lebot V (2006) Nature of taro (*Colocasia esculenta* (L.) Schott) genetic diversity prevalent in a Pacific Ocean island, Vanua Lava, Vanuatu. *Genet Resour Crop Ev* 53: 1273-1289, doi:10.1007/s10722-005-3877-x.

17 Shigeta M (1990) Folk *in situ* conservation of ensete [*Ensete ventricosum* (Welw.) E.E. Cheesman]: toward the interpretation of indigenous agricultural science of the Ari, Southwestern Ethiopia. *African Study Monographs* 10: 93-107.

18 Lempiainen T (1989) Germination of the Seeds of Ulluco (*Ullucus tuberosus*, Basellaceae). *Econ Bot* 43: 456-463, doi: 10.1007/Bf02935918.

19 Malice N (2009) *Genetic Diversity and Structure of Three Andean Tubers: Oxalis tuberosa Molina, Ullucus tuberosus Caldas and Tropaeolum tuberosum Ruiz & Pav.* Ph.D. thesis, Gembloux Agricutural University, Belgium.

20 Rousi A *et al.* (1989) Morphological variation among clones of Ulluco (*Ullucus tuberosus*, Basellaceae) collected in southern Peru. *Econ Bot* 43: 58-72, doi: 10.1007/Bf02859325.

21 Malice M, Vogt CLV, Pissard A, Arbizu C, Baudoin JP (2009) Genetic diversity of the Andean tuber crop species *Ullucus tuberosus* as revealed by molecular (ISSR) and morphological markers. *Belgian Journal of Botany* 142: 68–82.
